# The Effect of Fructose and FTO Gene Polymorphism on the Efficacy of GLP-1 and GIP Combination Therapies: A Narrative Review

**DOI:** 10.7759/cureus.110199

**Published:** 2026-06-03

**Authors:** Douglas S Larner

**Affiliations:** 1 Integrative Medicine, American College of Health Sciences, Portland, USA

**Keywords:** fructose, fto, gip, glp-1, insulin resistance, leptin, metabolic syndrome, obesity

## Abstract

This literature review examines how dietary fructose intake and fat mass and obesity-associated (*FTO*) gene polymorphisms may influence the efficacy of glucagon-like peptide-1 (GLP-1)- and glucose-dependent insulinotropic polypeptide (GIP)-based therapies in obesity and metabolic syndrome. The narrative review synthesizes evidence showing that incretin-based therapies, particularly GLP-1 receptor agonists and dual GLP-1/GIP agonists, produce substantial weight loss and metabolic improvement, but response varies considerably among individuals. A central theme is that high fructose consumption promotes hepatic steatosis, de novo lipogenesis, insulin resistance, oxidative stress, and systemic inflammation, thereby creating a metabolic environment that may contribute to variability in incretin responsiveness. In parallel, *FTO* risk alleles are associated with increased obesity susceptibility, altered appetite regulation, impaired leptin signaling, and greater vulnerability to metabolic dysfunction. The review proposes an integrated model in which *FTO*-mediated leptin resistance and fructose-induced hepatic and inflammatory stress converge to reduce the appetite-suppressive and metabolic benefits of GLP-1/GIP therapies. Furthermore, it discusses the clinical implications of this interaction, emphasizing dietary fructose reduction, combination pharmacotherapy, and precision medicine strategies such as *FTO* genotyping and individualized therapeutic selection. Overall, the evidence suggests that gene-diet interactions may meaningfully shape incretin treatment outcomes and that more personalized, multi-targeted approaches may improve weight-loss efficacy in patients with obesity and metabolic syndrome. However, direct clinical evidence linking *FTO* genotype and fructose intake to reduced GLP-1/GIP therapeutic efficacy remains limited.

## Introduction and background

Obesity has emerged as one of the most significant public health challenges of the twenty-first century. Since 1975, global obesity prevalence has nearly tripled, and more than 650 million adults worldwide are currently affected [1]. Among working-age adults, obesity contributes substantially to healthcare costs, reduced productivity, and increased risk of chronic diseases, including type 2 diabetes mellitus, cardiovascular disease, and metabolic dysfunction-associated steatotic liver disease (MASLD). Obesity develops through complex interactions among genetic susceptibility, dietary habits, environmental influences, and hormonal regulation of appetite and energy balance [2]. Understanding these interactions is essential for developing more effective and individualized treatment strategies.

One of the most important genetic factors associated with obesity is the fat mass and obesity-associated (*FTO*) gene. Variations within this gene, known as *FTO* polymorphisms, have been linked to increased appetite, higher energy intake, greater adiposity, and an elevated risk of obesity. Individuals carrying certain* FTO* risk alleles may also be more susceptible to leptin resistance, a condition in which the hormone leptin becomes less effective at signaling satiety and regulating body weight. At the same time, dietary factors, particularly excessive fructose consumption from sugar-sweetened beverages and processed foods, have been implicated in metabolic dysfunction through mechanisms involving hepatic lipogenesis, insulin resistance, hyperuricemia, and alterations in appetite regulation.

Recent advances in obesity pharmacotherapy have transformed treatment options through the development of incretin-based therapies. Glucagon-like peptide-1 (GLP-1) receptor agonists mimic the actions of the naturally occurring GLP-1 hormone, which promotes satiety, delays gastric emptying, and improves glucose regulation. More recently, dual agonists targeting both GLP-1 and glucose-dependent insulinotropic polypeptide (GIP) receptors have demonstrated even greater weight-loss efficacy. A comprehensive meta-analysis by Wong et al. involving 47 randomized controlled trials and 23,244 participants reported that GLP-1 receptor agonists produced a mean weight reduction of 4.57 kg compared with placebo, accompanied by significant reductions in body mass index (BMI) and waist circumference [3].

Despite these impressive outcomes, substantial variability exists in individual treatment response. Some patients experience dramatic and sustained weight loss, whereas others achieve only modest benefit. Emerging evidence suggests that this variability may be influenced by genetic factors, dietary patterns, and underlying metabolic dysfunction. Lifestyle interventions combined with GLP-1 receptor agonist therapy produce significantly greater weight loss and cardiometabolic improvements than lifestyle modification alone [4]; however, the biological factors that determine treatment responsiveness remain incompletely understood.

The mechanisms underlying this variability are likely multifactorial and may involve interactions among *FTO* gene polymorphisms, fructose-induced metabolic alterations, leptin signaling pathways, and incretin hormone physiology. Understanding how GLP-1 and GIP regulate appetite, energy expenditure, and glucose metabolism, and how these pathways may be modified by genetic and dietary factors, could help explain differences in therapeutic outcomes among individuals with obesity.

Therefore, the purpose of this narrative review is to examine the current evidence regarding the relationships among *FTO *gene polymorphisms, dietary fructose consumption, leptin resistance, and responsiveness to GLP-1/GIP-based therapies in adults with obesity. Rather than establishing a direct causal relationship, this review seeks to synthesize existing mechanistic and clinical evidence, identify potential pathways linking these factors, and generate hypotheses that may guide future research and personalized obesity treatment strategies.

## Review

Methods

This narrative review examines the interaction between GLP-1 and GIP-based therapies, fructose metabolism, and *FTO* gene polymorphisms in obesity and metabolic dysfunction. The clinical implications of these interacting pathways will be discussed, including dietary fructose restriction, combination pharmacotherapy, precision medicine strategies, and future directions for improving treatment outcomes.

Study design

This study was conducted as a hypothesis-generating narrative review. The purpose of the review was not to perform a systematic evidence synthesis or quantitative meta-analysis but rather to integrate findings from the fields of obesity medicine, nutrigenomics, endocrinology, and metabolic disease to develop a conceptual framework describing how dietary fructose intake and *FTO* gene polymorphisms may influence the efficacy of GLP-1 and GIP-based therapies.

Literature identification and selection

Relevant literature was identified through targeted searches of peer-reviewed publications focusing on GLP-1 receptor agonists, dual GLP-1/GIP agonists, fructose metabolism, obesity genetics, *FTO* polymorphisms, leptin resistance, insulin resistance, metabolic syndrome, and precision nutrition. Priority was given to recent systematic reviews, meta-analyses, randomized controlled trials, mechanistic studies, and landmark investigations that contributed substantially to understanding the physiologic and molecular pathways linking these topics. Additional references were identified through citation tracking of key publications.

Data synthesis

Evidence was synthesized narratively to examine potential biologic interactions among fructose metabolism, *FTO*-mediated obesity susceptibility, leptin signaling, and incretin-based pharmacotherapy. Findings from human clinical studies, translational investigations, and relevant animal studies were integrated to identify recurring mechanistic themes and areas of convergence. Particular emphasis was placed on pathways involving hepatic steatosis, de novo lipogenesis (DNL), systemic inflammation, insulin resistance, appetite regulation, and neuroendocrine control of energy balance.

Hypothesis development

The primary objective of this review was to generate and refine a mechanistic hypothesis rather than establish causality. The proposed model suggests that high fructose consumption and *FTO* risk alleles may converge through shared pathways involving leptin resistance, hepatic metabolic dysfunction, and chronic inflammation, thereby attenuating the appetite-suppressive and metabolic effects of GLP-1/GIP-based therapies. The conceptual framework presented should be considered exploratory and intended to guide future mechanistic investigations, prospective clinical studies, and precision-medicine approaches to obesity treatment. The key evidence supporting this proposed interaction between fructose intake, *FTO* polymorphisms, and GLP-1/GIP therapy response is summarized in Table 1.

**Table 1:** Summary of key evidence supporting the proposed interaction between fructose intake, FTO polymorphisms, and GLP-1/GIP therapy response FTO, fat mass and obesity-associated; GIP, glucose-dependent insulinotropic polypeptide; GLP-1, glucagon-like peptide-1

Evidence Domain	Main Findings	Potential Relevance to GLP-1/GIP Response
GLP-1 receptor agonists and weight loss	GLP-1 receptor agonists produce substantial weight loss primarily through appetite suppression and reduced caloric intake.	Establishes the primary mechanism through which incretin therapies induce weight loss.
Dual GLP-1/GIP agonists	Dual agonists demonstrate greater weight loss and metabolic improvement than selective GLP-1 receptor agonists.	Suggests that targeting multiple metabolic pathways may overcome resistance to therapy.
Fructose metabolism	Fructose undergoes unregulated hepatic metabolism, promoting de novo lipogenesis, hepatic steatosis, oxidative stress, and uric acid production.	Creates a metabolic environment that may impair responsiveness to incretin therapies.
Fructose and insulin resistance	High fructose intake contributes to hepatic and systemic insulin resistance independent of weight gain.	Insulin resistance may limit metabolic improvements achieved through GLP-1 signaling.
Fructose and leptin signaling	Fructose reduces postprandial insulin and leptin responses while impairing satiety signaling.	Reduced leptin sensitivity may diminish appetite-suppressive effects of GLP-1 therapies.
Gut microbiome alterations	High fructose intake promotes dysbiosis and intestinal permeability, while resistant starch improves microbial diversity and SCFA production.	Gut-derived inflammation may contribute to impaired metabolic signaling and reduced therapeutic efficacy.
*FTO* polymorphisms and obesity risk	*FTO* risk alleles are associated with increased obesity susceptibility, elevated appetite, and greater energy intake.	May predispose individuals to reduced weight-loss responsiveness.
*FTO *and leptin resistance	*FTO* risk alleles contribute to impaired hypothalamic leptin signaling and dysregulated appetite control.	May reduce the effectiveness of GLP-1-mediated appetite suppression.
Metabolic syndrome and GLP-1 efficacy	Individuals with insulin resistance and leptin resistance appear to experience reduced therapeutic responses.	Supports the hypothesis that advanced metabolic dysfunction attenuates incretin efficacy.

GLP-1 and GIP physiology: central and peripheral mechanisms of action in weight regulation

GLP-1 is an incretin hormone secreted by enteroendocrine L-cells in response to nutrient ingestion that plays a central role in regulating glucose homeostasis by stimulating glucose-dependent insulin secretion from pancreatic β-cells, suppressing glucagon release from α-cells, delaying gastric emptying, and reducing appetite via hypothalamic signaling. In the hypothalamus, GLP-1 receptor signaling modulates appetite-regulating neurons and the integration of energy balance signals, with central GLP-1 receptor activation suppressing feeding through effects on neuropeptide Y/agouti-related peptide and pro-opiomelanocortin neurons. Current evidence indicates that appetite suppression represents the principal mechanism underlying GLP-1 receptor agonist-induced weight loss. Both central and peripheral GLP-1 signaling pathways contribute to reduced energy intake, and clinical studies have consistently shown that the extent of weight loss correlates closely with decreases in appetite and caloric consumption [5].

GIP, also known as glucose-dependent insulin-releasing polypeptide, represents an important co-regulatory hormone that modulates both metabolic and behavioral aspects of energy homeostasis. Qiao et al. extensively reviewed the therapeutic potential of GIP, demonstrating that dual GLP-1/GIP receptor agonists show enhanced efficacy compared to selective GLP-1 receptor agonists, achieving greater weight loss and superior HbA1c reduction, with emerging evidence suggesting improved outcomes in MASLD [6]. The superior efficacy of dual agonists likely reflects the synergistic effects of GIP on metabolic homeostasis; GIP modulates bone remodeling, reduces ectopic fat deposition, and influences cardiovascular lipid metabolism. Beyond appetite suppression, GLP-1 receptor agonists exert metabolic benefits that contribute to improved cardiometabolic health independent of weight loss, as demonstrated by multiple mechanisms including improved lipid profiles and vascular effects.

Dietary factors significantly influence postprandial GLP-1 secretion patterns, with fructose ingestion paradoxically triggering GLP-1 secretion through intestinal L-cell activation despite its unique hepatic metabolic fate. Murao et al. discovered that intestinal fructose metabolism triggers a GLP-1-β-cell axis to prevent post-fructose hyperglycemia, demonstrating that fructose ingestion directly stimulates GLP-1 secretion through L-cell activation via fructolysis and ATP/ADP (adenosine triphosphate/adenosine diphosphate) ratio changes [7]. This counterintuitive finding suggests that fructose metabolism in intestinal L-cells directly stimulates GLP-1 secretion, which then attempts to potentiate insulin secretion and counteract fructose-induced hyperglycemia. However, this compensatory GLP-1 response is paradoxically overwhelmed by fructose's direct pathogenic effects on metabolic homeostasis, creating a scenario where elevated GLP-1 levels fail to overcome the metabolic dysfunction induced by fructose consumption.

Fructose metabolism: distinct pathways, metabolic consequences, and interference with metabolic homeostasis

Fructose undergoes fundamentally different metabolic processing compared to glucose, with profound implications for metabolic health and the development of metabolic syndrome in adults consuming high-fructose diets. Unlike glucose, which is distributed throughout the body after intestinal absorption, fructose is primarily extracted by the liver through extensive first-pass hepatic extraction. Park et al. comprehensively documented the mechanisms of fructose and hepatic insulin resistance, demonstrating that the liver represents the predominant site of fructose metabolism, where it is rapidly phosphorylated by ketohexokinase (KHK), the rate-limiting enzyme in fructolysis, which uniquely bypasses phosphofructokinase (PFK) regulation [8]. This unregulated metabolism triggers multiple pathological cascades; fructose metabolism produces glucose, lactate, triglycerides, free fatty acids, uric acid, and methylglyoxal, while ATP depletion during fructolysis induces oxidative stress and inflammatory responses that disturb hepatic and systemic functions.

Fructose uniquely promotes DNL compared to glucose, with profound metabolic consequences that establish an environment hostile to GLP-1 efficacy. Stanhope et al. demonstrated in a landmark study that consuming fructose-sweetened, not glucose-sweetened, beverages increases visceral adiposity and lipids and decreases insulin sensitivity in overweight/obese humans [9]. Fructose consumption reduces fatty acid oxidation through the overproduction of malonyl-CoA, a potent inhibitor of carnitine palmitoyltransferase-1 (CPT-1), thereby preventing fatty acid entry into mitochondria for oxidative metabolism. A critical distinction between fructose and glucose is that fructose does not stimulate insulin or leptin secretion to the degree that glucose does. Dietary fructose reduces circulating insulin and leptin, attenuates postprandial suppression of ghrelin, and increases triglycerides in women, demonstrating that this diminished leptin response is particularly concerning because leptin is a critical satiety signal regulating long-term energy balance, and its suppression with chronic fructose consumption leads to increased caloric intake and weight gain [10].

High-fructose consumption triggers systemic inflammation and oxidative stress through multiple interconnected pathways that establish a pro-inflammatory metabolic environment actively antagonistic to GLP-1 signaling. DNL generates reactive oxygen species through mitochondrial stress, while impaired mitochondrial β-oxidation capacity leads to lipid overload and ectopic fat deposition. Coronati et al. presented a comprehensive narrative review of added fructose in non-alcoholic fatty liver disease and metabolic syndrome, highlighting how fructose consumption alters gut microbiota composition, disrupts the integrity of the intestinal epithelial barrier, and promotes lipopolysaccharide translocation, which activates toll-like receptor 4 (TLR4)-mediated inflammation [11]. The resulting metabolic endotoxemia drives systemic IL-6 and TNF-α production, further impairing leptin and insulin signaling. A systematic analysis of insulin production and resistance in different models of diet-induced obesity and metabolic syndrome demonstrated that fructose induces hepatic insulin resistance through multiple mechanisms that persist independent of weight gain, creating a fundamental barrier to metabolic improvement through reduced insulin receptor substrate-2 expression and increased protein-tyrosine phosphatase 1B activity [12].

*FTO* gene polymorphism and leptin resistance in obesity susceptibility

The *FTO* gene represents the most consistently replicated common genetic variant associated with BMI across diverse populations, with particular relevance for understanding obesity susceptibility in adults across all age groups. Shahid et al. demonstrated that the common variant of *FTO *gene rs9939609 was significantly associated with obesity in Pakistani females, with the risk allele showing consistent associations with increased BMI, fat mass, and increased energy intake across pediatric and adult populations [13]. The most well-studied polymorphism, rs9939609 (T/A), shows strong associations with obesity; individuals homozygous for the risk allele (AA) have approximately 1.6-fold increased obesity risk compared to TT homozygotes [14]. Furthermore, *FTO *rs17817449 variant was associated with an increased risk of severe obesity in a Brazilian cohort in their case-control study, showing that distinct cytokine expression patterns are observed across *FTO* rs17817449 genotypes, suggesting that *FTO* variants influence both metabolic and inflammatory aspects of obesity [14].

The *FTO* gene encodes an N6-methyladenosine (m6A) RNA demethylase that regulates post-transcriptional gene expression through m6A modification, influencing multiple biological processes critical to metabolic regulation. Fredriksson et al. established that the obesity gene *FTO* is of ancient origin, up-regulated during food deprivation and expressed in neurons of feeding-related nuclei of the brain, providing foundational evidence that *FTO *is highly expressed in feeding-related brain nuclei, particularly the hypothalamic arcuate nucleus, paraventricular nucleus, and dorsomedial hypothalamus [15]. *FTO* expression is dynamically regulated; it is upregulated during fasting, suggesting roles in energy homeostasis and feeding behavior initiation. Moreover, there is a link between *FTO*, ghrelin, and impaired brain food-cue responsivity, showing that carriers of the *FTO* risk allele (AA and AT genotypes) demonstrate altered ghrelin signaling, with dysregulation of circulating acyl-ghrelin levels and impaired postprandial appetite reduction [16]. For adults carrying *FTO* risk alleles, this genetic architecture creates elevated baseline appetite signaling and reduces satiety responsiveness, establishing a metabolic predisposition toward increased food intake and weight gain.

The mechanisms by which *FTO* variation contributes to obesity involve profound effects on leptin signaling and hypothalamic leptin sensitivity that directly antagonize GLP-1's appetite-suppressive effects. The hypothalamic *FTO* promotes high-fat diet-induced leptin resistance in mice through increasing CX3CL1 expression, showing that the *FTO* risk allele (A) promotes leptin resistance through m6A-mediated post-transcriptional regulation of downstream effectors [17]. High-fat diet feeding in mice increases *FTO* expression in the hypothalamus, which triggers upregulation of chemokine ligand 1 and suppressor of cytokine signaling 3 through m6A methylation and demethylation of target mRNAs, resulting in impaired JAK2-STAT3 signaling in leptin-responsive neurons and effectively creating hypothalamic leptin resistance. Magno et al. examined the influence of *FTO* rs9939609 polymorphism on appetite, ghrelin, leptin, IL6, TNFα levels, and food intake of women with morbid obesity, demonstrating that *FTO* risk allele carriers show dysregulated responses in brain regions controlling appetite, processing, and incentive motivation. The net effect is that *FTO* risk allele carriers possess both elevated ghrelin (appetite-stimulating) and impaired leptin signaling (appetite-suppressing), creating a fundamental metabolic state predisposed to increased food intake and weight gain [18].

*FTO* genetic susceptibility, fructose consumption, and GLP-1/GIP dysfunction in metabolic syndrome

The convergence of *FTO *genetic susceptibility, high fructose consumption, and impaired GLP-1 responsiveness creates a unified mechanism for severe metabolic syndrome development and compromised GLP-1 receptor agonist efficacy. For the purposes of this review, severe metabolic syndrome is defined as the presence of metabolic syndrome according to established diagnostic criteria, accompanied by clinically significant insulin resistance, central obesity, and evidence of advanced metabolic dysfunction, such as MASLD, elevated inflammatory biomarkers, impaired glucose regulation, and marked leptin resistance. Farzand et al. comprehensively reviewed nutrigenomics of obesity by integrating genomics, epigenetics, and diet-microbiome interactions for precision nutrition, emphasizing how individuals carrying *FTO* risk alleles (particularly AA and AT genotypes) start with genetic predisposition to hypothalamic leptin resistance and elevated appetite signaling [19]. When these genetically susceptible individuals consume high amounts of fructose, their hepatic metabolism becomes saturated with fructose-derived metabolites, triggering hepatic steatosis, mitochondrial dysfunction, and inflammatory signaling. The resulting hepatic and systemic inflammation, combined with endoplasmic reticulum stress, impairs both hepatic and central nervous system insulin and leptin signaling, creating a compounded state of resistance to appetite-suppressive hormone signaling.

**Figure 1:**
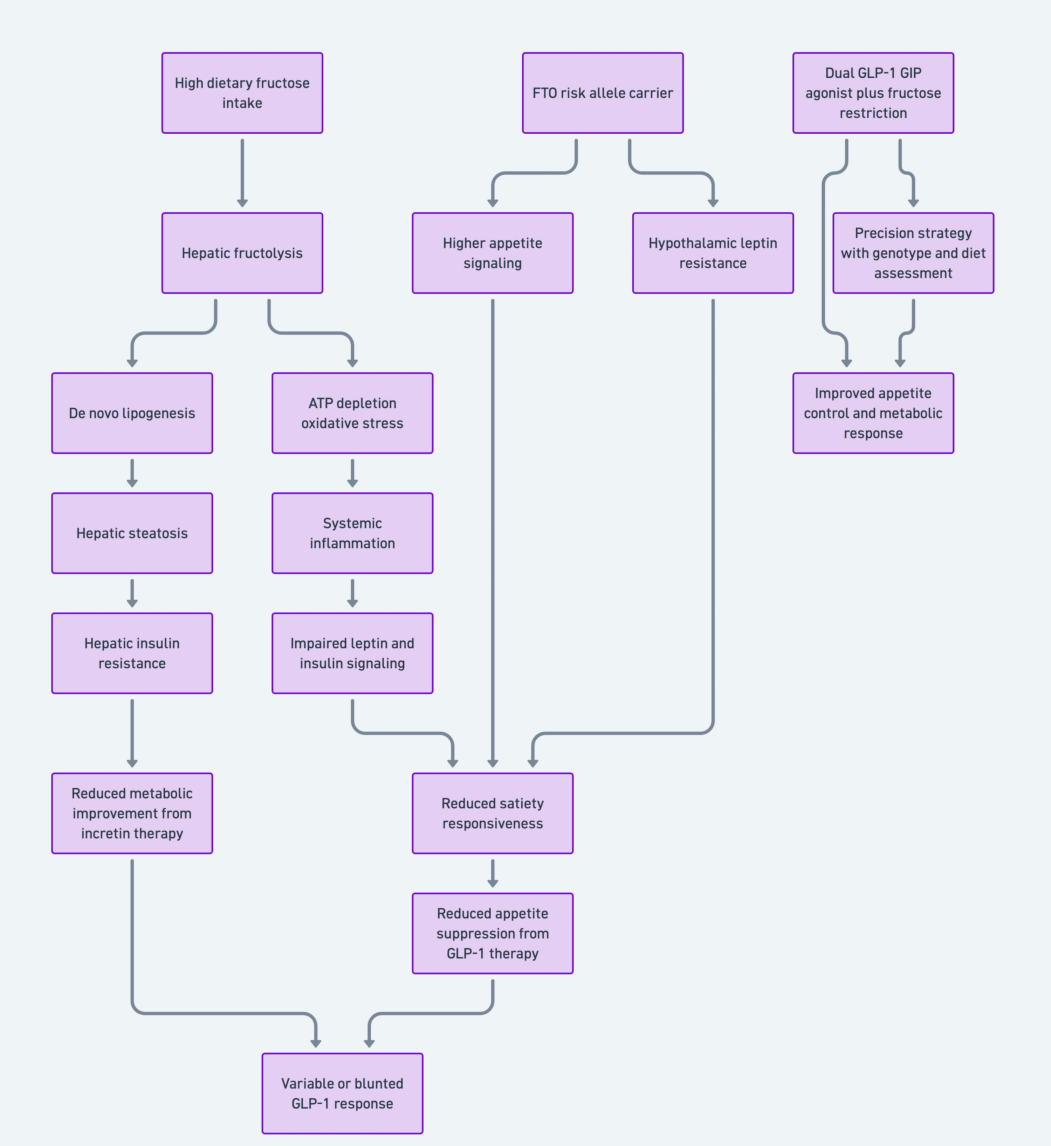
Integrated model of fructose intake, FTO genetic susceptibility, leptin resistance, and reduced GLP-1/GIP therapeutic response The figure was developed using Adobe software (No AI was used in the development of the referenced figure). ATP, adenosine triphosphate; FTO, fat mass and obesity-associated; GIP, glucose-dependent insulinotropic polypeptide; GLP-1, glucagon-like peptide-1

This integrative metabolic dysfunction manifests clinically as resistance to GLP-1 receptor agonist therapy through multiple coordinated mechanisms that establish a metabolic ceiling limiting therapeutic efficacy. Evidence suggests that GLP-1 receptor agonists effectively reduce body weight in individuals with preserved metabolic function and intact leptin signaling. However, therapeutic efficacy appears to be attenuated in individuals with established metabolic syndrome, characterized by leptin resistance and insulin resistance, indicating that underlying metabolic dysfunction may limit responsiveness to incretin-based therapies [20]. The proposed mechanism is that leptin resistance limits GLP-1's ability to access and activate hypothalamic appetite-regulatory circuits; severely elevated and dysregulated leptin levels impair leptin receptor signaling and downstream STAT3 activation, which is necessary for GLP-1-mediated appetite suppression. Additionally, fructose-induced hepatic insulin resistance creates a ceiling effect, preventing further improvement in hepatic insulin extraction despite elevated GLP-1 levels, as demonstrated by Keyhani-Nejad et al., who showed that endogenously released GIP reduces and GLP-1 increases hepatic insulin extraction through distinct mechanisms [21]. High dietary fructose promotes hepatic fructolysis, DNL, oxidative stress, inflammation, hepatic steatosis, and insulin resistance. In patients with *FTO* risk alleles, elevated appetite signaling and hypothalamic leptin resistance may further reduce satiety responsiveness. Together, these pathways may contribute to variable or blunted response to GLP-1-based therapy, supporting combined dietary and precision pharmacologic strategies [19-21] (Figure 1).

Evidence from metabolic pathway analyses reveals that *FTO*-mediated effects on lipogenic enzyme activity are dramatically amplified when combined with high fructose intake, creating particularly severe metabolic dysfunction. Başaranoğlu established fructose as a key player in the development of fatty liver disease, demonstrating that *FTO* function is directly linked to DNL and fatty acid metabolism [22]. When hepatic fatty acid synthase expression is downregulated in *FTO* knock-out mice, *FTO* inhibition reduces hepatic triglyceride synthesis, whereas *FTO* overexpression in hepatocytes increases lipogenic gene expression, suggesting that *FTO* genotype influences susceptibility to fructose-induced hepatic steatosis. Individuals with *FTO *risk alleles (AA genotype) likely have constitutively elevated lipogenic enzyme activity, meaning that when challenged with high fructose intake, which itself massively stimulates DNL through hyperactivation of SREBP-1c and other lipogenic transcription factors, their hepatic lipogenic capacity becomes maximally engaged. This creates a perfect storm for rapid hepatic fat accumulation, development of metabolic dysfunction, and establishment of conditions that actively oppose the beneficial effects of GLP-1 receptor agonist therapy [22].

Evidence of GLP-1 resistance in *FTO*-positive, fructose-consuming individuals

The integrated pathway of *FTO*-fructose-leptin resistance-GLP-1 dysfunction explains several clinically observed patterns in metabolic disease that demonstrate how genetic and dietary factors converge to limit therapeutic efficacy. Taskinen et al. reviewed dietary fructose and the metabolic syndrome, demonstrating that the association between high-fructose diet consumption and the prevalence of metabolic syndrome is stronger than the association with high-fat diet consumption alone [23]. Huber et al. examined the dietary impact on fasting and stimulated GLP-1 secretion in different metabolic conditions through a narrative review, showing that individuals with metabolic syndrome show impaired GLP-1 responses to mixed meals despite elevated fasting GLP-1 levels, suggesting that chronic impairment of GLP-1 receptor signaling occurs in the context of leptin resistance [24]. *FTO* risk allele carriers preferentially accumulate visceral adiposity, with serum spexin, adiponectin, and leptin levels in polycystic ovarian syndrome associated with *FTO* gene polymorphism, producing particularly inflammatory adipokines that amplify leptin resistance and further impair GLP-1 signaling [25]. The combination of high fructose consumption and *FTO* risk allele carriage particularly predicts severe phenotypes of nonalcoholic fatty liver disease/metabolic dysfunction-associated steatohepatitis (NAFLD/MASH), including greater hepatic inflammation and fibrosis.

Adults carrying *FTO* risk alleles and consuming high fructose demonstrate reduced responsiveness to GLP-1 receptor agonist monotherapy through mechanisms that limit both central and peripheral GLP-1 action. de Soysa et al. examined the *FTO *gene allele rs9939609 and its associations with glucose tolerance, hepatic insulin sensitivity, and total insulin sensitivity in adults with obesity and found that while the *FTO* risk allele is associated with greater baseline obesity, the percentage weight loss achieved through structured programs is comparable between genotype groups [26]. This suggests that the fundamental capacity for weight loss exists but is constrained by specific metabolic barriers rather than absolute genetic determinism. However, the pathway of weight loss differs by genotype; *FTO* risk allele carriers show greater reductions in leptin levels, reflecting greater leptin resistance reversal relative to fat mass reduction, suggesting that *FTO*-mediated effects on leptin biology may explain variable weight loss responses across individuals and that combined therapies addressing multiple targets may be necessary to achieve sustained metabolic improvement.

A crucial clinical implication of this integrated model is that individuals with metabolic syndrome who carry *FTO* risk alleles and consume high amounts of fructose demonstrate reduced responsiveness to conventional GLP-1 receptor agonist therapy. Moiz et al. presented evidence on the comparative efficacy of GLP-1 receptor agonists and co-agonists for weight loss among patients without diabetes through a network meta-analysis showing that while GLP-1 receptor agonists effectively reduce body weight in lean individuals and in people without leptin resistance, their efficacy is diminished in people with established metabolic syndrome [27]. Furthermore, combined in-person and home-based circuit exercise improved body composition and hormonal profiles in patients with post-bariatric weight regain through a genotype-stratified single-arm interventional study, showing that patients carrying *FTO* risk alleles who regained weight after bariatric surgery demonstrated differential responses [28]. This suggests that *FTO-*mediated effects on leptin biology may explain variable weight loss responses across individuals and supports the rationale for combined therapies addressing multiple targets.

Dietary intervention, combination pharmacotherapy, and precision medicine approaches

Given the central role of fructose in dampening GLP-1 benefits and amplifying *FTO*-mediated obesity susceptibility, dietary fructose restriction emerges as a critical foundational therapy for metabolic syndrome management in adults, particularly those with *FTO* risk alleles. Clinical and preclinical evidence consistently demonstrates that reducing fructose intake improves metabolic parameters independent of overall caloric intake. Dietary substitution of fructose with whole-grain carbohydrates containing resistant starch has been associated with beneficial shifts in gut microbiota composition, including enrichment of Bacteroidetes and increased short-chain fatty acid production, which may help mitigate metabolic dysfunction and hyperuricemia associated with metabolic syndrome [29]. Kuo et al. discovered that fermented black soybean and dehulled adlay improve metabolic syndrome via AMPK-SIRT1 activation and gut microbiota modulation, supporting the approach of dietary modification to reverse the metabolic dysfunction induced by high fructose consumption [30]. The replacement of sugar-sweetened beverages with other non-caloric alternatives has emerged as a particularly effective intervention point; Li et al. identified the role of sugar-sweetened beverages in the progression of a murine MASLD model, demonstrating that fructose uniquely accelerates MASLD progression [31].

For individuals with established metabolic syndrome who are carriers of *FTO *risk alleles, combination therapies addressing multiple pathophysiologic mechanisms show particular promise in overcoming the metabolic ceiling effects observed with GLP-1 monotherapy. de Ceglia et al. in their research on a combined GLP-1/PPARα/CB1-based therapy to restore the central and peripheral metabolic dysregulation induced by a high-fructose high-fat diet demonstrated that this combined approach showed superior efficacy compared to single agents [32]. The combined therapy reversed both peripheral metabolic alterations (body weight, plasma triglycerides, hepatic parameters) and central alterations (disrupted food intake regulation, altered insulin pathway signaling), demonstrating that dual or triple agonist therapy may overcome the leptin resistance and hepatic insulin resistance that limits single-agent GLP-1 receptor agonist efficacy.

Additionally, emerging evidence supports combining GLP-1 receptor agonists with targeted approaches to restore leptin sensitivity, representing a rational strategy for refractory metabolic dysfunction. Ruchko et al. examined β-cell heterogeneity and molecular plasticity in type 2 diabetes from multi-omics perspectives and the role of gut microbiota, providing insights into how combination therapies might synergize with GLP-1 therapy to overcome leptin resistance [33]. Multiple researchers investigated foundational principles of fructose, insulin resistance, and metabolic dyslipidemia, demonstrating that direct JAK2 activators, SOCS3 inhibitors, or approaches enhancing leptin transport across the blood-brain barrier might synergize with GLP-1 therapy to overcome leptin resistance [33,34]. Furthermore, emerging evidence supports the role of indicating sugar, uric acid, and fructose metabolism in diabetes and obesity, supporting the rationale for KHK inhibitors, which block the rate-limiting enzyme of fructose metabolism, as a rational therapeutic approach to prevent fructose-induced metabolic dysfunction [34]. The combination of GLP-1 receptor agonist pharmacotherapy with structured dietary fructose reduction addresses both the genetic susceptibility (through reduced engagement of *FTO*-mediated lipogenic pathways) and the environmental driver (fructose-induced metabolic dysfunction and leptin resistance).

Future research directions: *FTO* genotyping, dietary fructose exposure, and precision obesity therapy

The growing field of nutrigenomics has generated interest in whether genetic factors may contribute to the marked interindividual variability observed in response to obesity therapies. Among the most extensively studied obesity-related genes, the *FTO* gene has consistently been linked to increased adiposity, altered appetite regulation, greater energy intake, and differences in metabolic flexibility. Farzand et al. reported that individuals carrying *FTO* risk alleles demonstrate greater baseline obesity and differential responses to lifestyle interventions, although overall weight-loss capacity appears to remain preserved [19]. Similarly, Andreasen et al. demonstrated that low physical activity amplifies the effects of the *FTO* rs9939609 polymorphism on body fat accumulation, while subsequent large-scale meta-analyses have highlighted significant interactions between *FTO* genotype and environmental factors such as diet and physical activity [35,36].

These observations raise the possibility that the *FTO *genotype may contribute to heterogeneity in metabolic responses to incretin-based therapies. However, direct clinical evidence linking specific *FTO *variants to differential responsiveness to GLP-1 receptor agonists or dual GLP-1/GIP agonists remains limited. Consequently, *FTO *genotyping should currently be viewed as an investigational tool rather than a basis for routine therapeutic selection.

Several biologically plausible mechanisms support future investigation in this area. Mendelian randomization studies and mechanistic investigations suggest that *FTO* genetic variation influences appetite regulation, energy expenditure, DNL, and hepatic metabolic flexibility [37]. Individuals carrying *FTO* risk alleles may exhibit greater susceptibility to fructose-induced metabolic dysfunction because *FTO*-mediated alterations in lipogenic pathways can amplify hepatic triglyceride accumulation, oxidative stress, and inflammatory signaling when combined with high fructose exposure. These pathways converge on mechanisms implicated in insulin resistance, leptin resistance, and impaired appetite regulation, all of which have been proposed as potential determinants of incretin responsiveness.

Dietary fructose exposure may represent a particularly important environmental modifier of these genetic effects. High fructose intake promotes hepatic fructolysis, DNL, uric acid generation, oxidative stress, hepatic steatosis, and systemic inflammation. These metabolic disturbances contribute to impaired leptin and insulin signaling and may establish physiologic conditions that attenuate the effectiveness of GLP-1-mediated appetite suppression and metabolic regulation. Emerging evidence supporting the role of sugar, uric acid, and fructose metabolism in obesity and diabetes has also generated interest in KHK inhibitors, which target the rate-limiting enzyme of fructose metabolism and may represent a future therapeutic strategy for mitigating fructose-induced metabolic dysfunction [34].

Taken together, these observations suggest a potential conceptual framework in which genetic susceptibility (*FTO* risk alleles) and environmental exposure (high dietary fructose intake) interact to influence metabolic health and potentially modify response to incretin-based therapies. Future prospective studies should evaluate whether assessment of *FTO* genotype, fructose consumption patterns, leptin resistance, insulin resistance, hepatic steatosis, and inflammatory biomarkers can identify patient subgroups with distinct therapeutic responses.

Particularly important areas for future investigation include determining whether individuals with *FTO* risk alleles experience differential responses to selective GLP-1 receptor agonists versus dual GLP-1/GIP agonists, whether fructose restriction enhances treatment responsiveness in genetically susceptible individuals, and whether emerging therapies targeting fructose metabolism, such as KHK inhibitors, provide additive benefits when combined with incretin-based pharmacotherapy. Such studies may ultimately help establish whether genetic and dietary profiling can contribute to precision-medicine approaches for obesity management.

**Figure 2:**
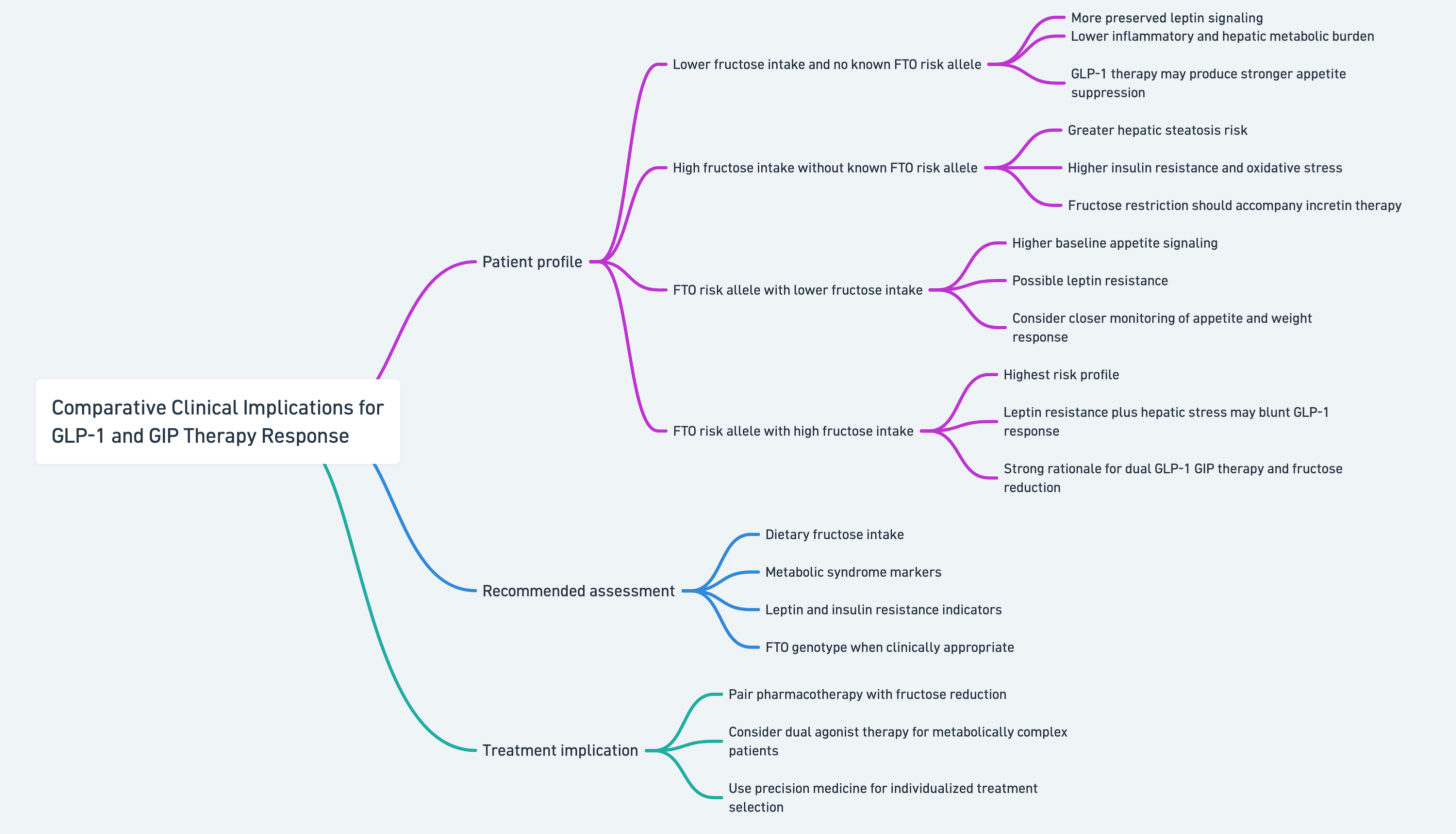
FTO genotyping, dietary assessment, and personalized therapeutic selection The figure was developed using Adobe software (No AI was used in the development of the referenced figure). FTO, fat mass and obesity-associated; GIP, glucose-dependent insulinotropic polypeptide; GLP-1, glucagon-like peptide-1

Until prospective clinical trials directly evaluate these relationships, the proposed interactions among *FTO* genotype, dietary fructose exposure, leptin resistance, and GLP-1/GIP responsiveness should be considered hypothesis-generating rather than evidence-based criteria for clinical decision-making. Figure 2 presents a conceptual model intended to guide future research exploring these potential genotype-environment-treatment interactions [38-39].

Outstanding questions and future research directions

While substantial evidence supports the integrated model of *FTO*-mediated genetic susceptibility, fructose-induced leptin resistance, and GLP-1 dysfunction in metabolic syndrome development, critical mechanistic questions remain unresolved. Karimzadeh et al. examined the regulation of feeding and metabolism by fat mass and obesity-associated protein in zebrafish, establishing that the precise molecular mechanisms by which *FTO* demethylase activity on specific m6A-modified transcripts alters leptin signaling remain incompletely understood [40]. It remains unclear whether *FTO* directly methylates leptin receptor mRNA or downstream signaling molecules such as STAT3, or whether it works through indirect effects on transcription factor expression. Additionally, the tissue-specific roles of *FTO *remain unclear; while hypothalamic *FTO* clearly influences appetite regulation, the relative contributions of hepatic, adipose, and intestinal *FTO* to the overall metabolic phenotype require clarification through future studies.

Furthermore, the interplay between fructose-induced changes in gut microbiota and *FTO*-mediated leptin resistance deserves further investigation. Nutrient-induced remodeling of the adipose-cardiac axis with metabolic flexibility, adipokine signaling, and therapeutic implications for cardiometabolic disease raise important questions about whether *FTO* genotype influences the capacity of fructose to cause dysbiosis or whether fructose-induced microbiota changes occur independent of* FTO *genotype but then interact with genetic susceptibility [41]. Similarly, whether *FTO* influences the responsiveness of gut L-cells to fructose (determining the magnitude of GLP-1 secretion) requires direct investigation. Zilstorff et al. demonstrated that the secretion of glucagon, GLP-1, and GIP may be affected by circadian rhythm in healthy males, suggesting that circadian factors may also modulate the *FTO*-fructose-GLP-1 interaction [42].

Multiple clinical research priorities emerge from this analysis. First, longitudinal studies are needed to directly compare outcomes in *FTO* risk allele carriers consuming high-fructose versus low-fructose diets and treated with GLP-1 receptor agonist monotherapy versus combination therapies in order to determine whether the predicted genotype-environment-treatment interactions are borne out in clinical practice. Supporting the need for further mechanistic study, Timper et al. demonstrated that GLP-1 receptor signaling in astrocytes regulates fatty acid oxidation, mitochondrial integrity, and cellular function, highlighting central nervous system pathways that may influence therapeutic response [43]. Extending this metabolic framework across the lifespan, Galderisi et al. showed that fructose consumption contributes to hyperinsulinemia in obese adolescents through a GLP-1-mediated mechanism, suggesting that altered incretin responses may begin early in the course of metabolic disease. In parallel, German et al. emphasized the relevance of plausible genetic determinants of weight-loss response to GLP-1 receptor agonists and bariatric surgery, reinforcing the importance of precision medicine approaches. Broader therapeutic implications are also emerging: Apperloo et al. highlighted the cardiorenal protective potential of GLP-1-based therapies in metabolic disease, suggesting that combination strategies may provide benefits beyond weight reduction alone. Similarly, Shao et al. reviewed progress in oral GLP-1 receptor agonists for type 2 diabetes, raising the possibility that novel formulations may differ in how they interact with *FTO* genotype and fructose-related metabolic dysfunction [44-47]. Collectively, these findings support a mechanistic framework in which fructose-induced metabolic dysfunction and *FTO*-associated genetic susceptibility may interact to influence responsiveness to GLP-1/GIP-based therapies. The proposed pathways and their potential effects on therapeutic efficacy are summarized in Table 2.

**Table 2:** Proposed mechanisms by which fructose intake and FTO risk alleles may reduce GLP-1/GIP therapeutic efficacy FTO, fat mass and obesity-associated; GIP, glucose-dependent insulinotropic polypeptide; GLP-1, glucagon-like peptide-1

Factor	Mechanism	Metabolic Consequence	Potential Impact on GLP-1/GIP Therapy
High dietary fructose intake	Unregulated hepatic fructolysis	Increased de novo lipogenesis and hepatic fat accumulation	May reduce responsiveness to incretin therapies
High dietary fructose intake	ATP depletion and uric acid generation	Oxidative stress and mitochondrial dysfunction	Impairs metabolic flexibility and insulin sensitivity
High dietary fructose intake	Reduced insulin and leptin signaling	Increased hunger and reduced satiety	Opposes appetite-suppressive actions of GLP-1/GIP agonists
*FTO *risk alleles	Altered energy-regulating pathways	Increased obesity susceptibility and adiposity	Predisposes to reduced weight-loss responsiveness
*FTO* risk alleles	Impaired hypothalamic leptin signaling	Central leptin resistance	Reduces effectiveness of GLP-1-mediated appetite regulation
Leptin resistance	Impaired JAK2-STAT3 signaling	Reduced satiety signaling	Limits central appetite-suppressive effects
Insulin resistance	Altered glucose utilization	Hyperinsulinemia and worsening metabolic syndrome	Reduces metabolic response to therapy
Fructose + *FTO* interaction	Activation of lipogenic and inflammatory pathways	Accelerated obesity and MASLD progression	Associated with reduced incretin efficacy
Advanced metabolic syndrome	Combined leptin resistance, insulin resistance, steatosis, and inflammation	Severe metabolic dysfunction	May establish a therapeutic ceiling limiting weight loss
Fructose restriction and resistant starch	Improved gut microbiota composition	Reduced inflammation and improved insulin sensitivity	May enhance responsiveness to therapy

Limitations

As a narrative review, this work is subject to the inherent limitations of non-systematic evidence synthesis, including potential selection bias and heterogeneity among included studies. No formal risk-of-bias assessment, systematic search protocol, or quantitative pooling of results was performed. Consequently, conclusions should be interpreted as hypothesis-generating rather than definitive evidence of clinical effect.

## Conclusions

GLP-1/GIP receptor agonists are highly effective for weight loss, but their response may be influenced by *FTO* genetic risk, dietary fructose intake, and the presence of metabolic syndrome. In adults with *FTO* risk alleles, high fructose consumption, insulin resistance, and leptin resistance, the metabolic environment may reduce the appetite-regulating and metabolic benefits of incretin therapy. Patients who respond poorly to GLP-1 monotherapy may benefit from a broader strategy that includes dual GLP-1/GIP agonists, intensified lifestyle intervention, and emerging therapies targeting leptin sensitivity, hepatic fat accumulation, or fructose metabolism. Overall, sustained weight loss in metabolically complex patients will likely require integrated treatment strategies that combine nutrition, pharmacotherapy, and precision medicine rather than relying on incretin therapy alone.

The conceptual framework proposed in this review suggests that variability in GLP-1/GIP therapeutic response may be driven not only by differences in medication adherence or baseline obesity severity but also by the interaction between dietary fructose exposure, genetic susceptibility, and the degree of underlying metabolic dysfunction. Specifically, fructose-induced hepatic steatosis, chronic inflammation, insulin resistance, and leptin resistance may converge with *FTO*-mediated alterations in appetite regulation to create a metabolic environment that limits the full therapeutic potential of incretin-based therapies. This hypothesis highlights the importance of viewing obesity as a heterogeneous disease with distinct metabolic phenotypes rather than a uniform clinical condition. Future prospective studies evaluating fructose intake, *FTO* genotype, leptin sensitivity, and treatment response are needed to determine whether these factors can be used to identify patients most likely to benefit from specific incretin-based treatment strategies and to support the development of precision medicine approaches for obesity management.
